# Innovative dual system approach for selective eradication of cancer cells using viral-based delivery of natural bacterial toxin–antitoxin system

**DOI:** 10.1038/s41388-021-01792-8

**Published:** 2021-06-25

**Authors:** Shiran Shapira, Ilana Boustanai, Dina Kazanov, Marina Ben Shimon, Ahmad Fokra, Nadir Arber

**Affiliations:** 1 The Integrated Cancer Prevention Center and the Health Promotion Center, Tel Aviv Sourasky Medical Center, Tel Aviv-Yafo, Israel; 2 Sackler Faculty of Medicine, Tel Aviv University, Tel Aviv, Israel

**Keywords:** Gene therapy, Targeted therapies, Colorectal cancer, Pancreatic cancer, Lung cancer

## Abstract

The inactivation of p53, a tumor suppressor, and the activation of the RAS oncogene are the most frequent genetic alterations in cancer. We have shown that a unique *E. coli* MazF-MazE toxin–antitoxin (TA) system can be used for selective and effective eradication of *RAS*-mutated cancer cells. This out of the box strategy holds great promise for effective cancer treatment and management. We provide proof of concept for a novel platform to selectively eradicate cancer cells using an adenoviral delivery system based on the adjusted natural bacterial system. We generated adenoviral vectors carrying the mazF toxin (pAdEasy-Py4-SV40mP-mCherry-MazF) and the antitoxin mazE (pAdEasy-RGC-SV40mP-MazE-IRES-GFP) under the regulation of *RAS* and *p53*, resp. The control vector carries the toxin without the RAS-responsive element (pAdEasy-ΔPy4-SV40mP-mCherry-MazF). In vitro, the mazF-mazE TA system (Py4-SV40mP-mCherry-MazF+RGC-SV40mP-MazE-IRES-GFP) induced massive, dose-dependent cell death, at 69% compared to 19% for the control vector, in a co-infected HCT116 cell line. In vivo, the system caused significant tumor growth inhibition of HCT116 (*KRAS*^*mut*^*/p53*^*mut*^) tumors at 73 and 65% compared to PBS and ΔPY4 control groups, resp. In addition, we demonstrate 65% tumor growth inhibition in HCT116 (*KRAS*^*mut*^*/p53*^*wt*^) cells, compared to the other two control groups, indicating a contribution of the antitoxin in blocking system leakage in WT RAS cells. These data provide evidence of the feasibility of using mutations in the *p53* and *RAS* pathway to efficiently kill cancer cells. The platform, through its combination of the antitoxin (mazE) with the toxin (mazF), provides effective protection of normal cells from basal low activity or leakage of mazF.

## Introduction

In normal tissue, cell proliferation is a strictly regulated process. Normal tissue architecture and function rely on accurate cell number homeostasis. This homeostasis is a result of a dynamic balance between growth-promoting and growth-inhibiting signals [1]. The promoting signals are transduced mainly by growth factors that bind cell-surface receptors, commonly containing intracellular tyrosine kinase domains [2, 3]. Cancer cells may deregulate the balance and induce constant proliferative stimulation.

*RAS*, a proto-oncogene, is the driver mutation in some of the most common aggressive and lethal malignancies such as pancreatic, colorectal, and lung cancer (approx. 90%, 50%, and 35%, resp.) [4–6].

The mammalian *RAS* genes encode the proteins KRAS, NRAS, and HRAS, which function as molecular switches by cycling between GTP-bound active and GDP-bound inactive forms [7]. Normally, the initiating step of the RAS pathway is the binding of extracellular ligands to cognate tyrosine kinase receptors that recruit guanine nucleotide exchange factors (GEFs), which mediate RAS activation in the cytosol. Once RAS is activated, it proceeds to stimulate downstream effectors that eventually bind to the RAS-responsive DNA element (RRE) and induces the transcription of early response genes involved in cell survival, proliferation, and differentiation [8]. RAS signaling is regulated by a negative-feedback mechanism, mediated by intrinsic RAS GTPase activity that is activated by GTPase activating proteins (GAPs) [1, 7]. In malignant cells, the proto-oncogene RAS leads to the constitutive activation of downstream effectors due to a lack of GTPase activity [1, 8–10].

The well-known tumor suppressor gene *p53* is a transcription factor that is mutated in approximately 50% of all human cancers [11, 12]. p53 is activated by stress conditions such as DNA damage, oncogene activation, hypoxia, starvation, altered mitochondrial or ribosomal biogenesis, and denuded telomeres [13]. In normal cells, p53 protein levels are low due to a negative-feedback loop, which is regulated by murine/human double minute 2 gene (*MDM2*). MDM2 directs p53 to proteasome-mediated degradation. Cellular stress disrupts the MDM2–p53 interaction, leading to p53 stabilization, nuclear accumulation, and activation. Upon activation, p53 and its downstream target genes induce cell cycle arrest, allowing either repair and cell survival or, in case of irreversible damage, activation of senescence and apoptotic pathways [1, 13, 14]. In malignant cells, p53 loses its transcriptional activity most commonly due to missense mutations [14]. These occur mainly within the DNA-binding domain, leading to the impairment of its sequence-specific interactions with target gene promoters [14]. Although missense mutations are common, a considerable fraction of nonsense mutations gives rise to truncated inactive proteins [13]. In contrast, gain-of-function mutations may promote tumorigenic functions such as the induction of angiogenic factors, metastasis, resistance to specific therapies, and the inactivation of other members of the p53 family [14, 15].

We propose to take advantage of both the hyperactivated RAS pathway, as well as the WT p53 pathway, for the selective eradication of tumor cells while safe-guarding normal cells. In previous studies, we have successfully shown that the RRE, denoted as PY2 and consisting of Ets and AP-1-binding sites, is capable of selectively activating the expression of a destructive element called the p53 upregulated modulator of apoptosis (*PUMA*, kindly provided by Bert Vogelstein) that is able to kill cancer cells [16–19]. The RRE-activated cassette has been recently improved by replacing the killing agent PUMA with a very potent bacterial toxin, MazF. MazF is regulated by polyoma (PY) virus enhancer PY4, which consists of four repeats of the PY2 enhancer [19]. In nature, the ribonuclease activity of mazF is regulated by its antitoxin mazE, both of which are transcribed by the same operon within the *E. coli* chromosome [20, 21]. The PY2 system described above, uses the toxin–antitoxin (TA) system that is carefully and constantly balanced, depending on the activation stage of the RAS and p53 pathways [19]. We have previously shown that this strongly controlled bi-modular system is effective and specific in the elimination of pancreatic and colorectal cancer cells [19].

In this study, a precision cancer therapy is suggested, which can serve as a generic platform in cancer treatment, taking advantage of the genetic status of the tumor.

## Results

### The mazEF dual system mode of action

**Fig. 1:**
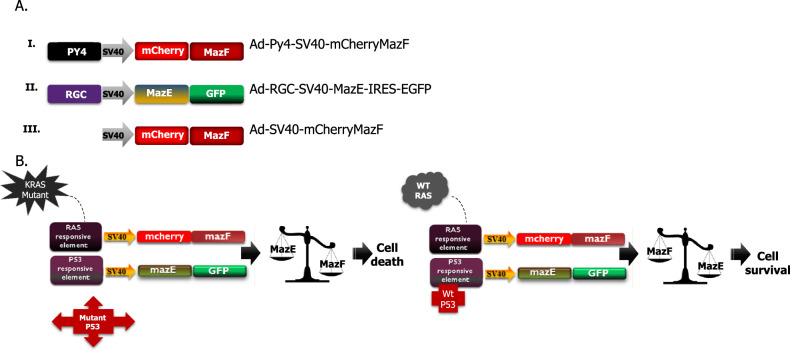
Schematic illustration of the toxin–antitoxin system. **A** Three adenovirus vectors were engineered: (i) mazF under the regulation of the RRE; (ii) mazE under the regulation of P53-responsive element; and (iii) control vector where the RRE was completely removed. **B** Model of the dual system-based mode of action in malignant and normal cells.

Three adenovirus vectors were engineered: (i) mazF under the regulation of the RAS-responsive element (RRE); (ii) mazE under the regulation of p53-responsive element; and (iii) MazF control vector where the RRE was completely removed (Fig. 1A). This dual system couples the ribonuclease activity of mazF with its antidote, mazE, with no transcription interference. Both genes are regulated according to the activation state of the oncogenic RAS and p53 pathways (Fig. 1B). This dual system is based on the balance between the activation of the destructive element and the activation of the neutralizing agent.

In cancer cells, mainly have both mutated RAS and p53, the downstream effectors of the activated RAS pathway bind to its responsive element (PY4) and induce the expression of the toxic agent.

However, the mutated p53 cannot bind to its RE; therefore, the levels of mazF will be much higher than those of mazE and these cells will die.

In normal cells, Ras and p53 are wt and therefore only basal levels of mazF will be detected. However, in these cells, the wt P53 binds to its RE therefore induces the expression of the antidote. In addition, the known stoichiometry balance shows that one molecule of antitoxin can bind and inhibit two molecules of the toxin. So in this case, the balance will tend to favor the neutralizing agent and these cells will be protected and survive.

Therefore, in cancer cells, the balance tends to favor the expression of mazF, while in normal cells, the expression of mazE is higher, providing protection to the cells.

### MazF efficiently kills cells with hyperactive RAS pathway

We used a previously described in vitro model system that consists of normal enterocytes derived from rat ileum (IEC18 cells), and their hyperactive KRAS transformed derivative (R1 cells) [16–18, 22].

**Fig. 2:**
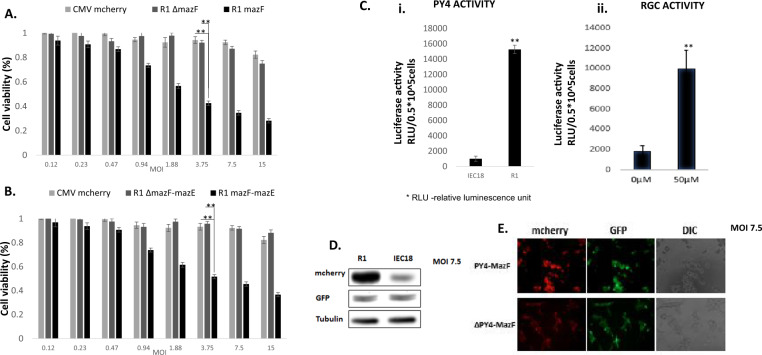
Eradication of R1 cells by recombinant toxin and antitoxin adenoviruses. **A** 1 × 10^4^ cells/well R1 cells were seeded onto 96-well plates and median dilutions of the toxin or the control viruses starting from an MOI of 15 were added to the cells. Cell survival was measured by the enzymatic MTT assay 72 h after infection and average values of triplicates from representative experiments were plotted. **B** Cells were treated with median dilutions of the mazEF or the ΔPY4-mazF-mazE control viruses as described above, starting from an MOI of 15. Cell survival was measured by the enzymatic MTT assay. **C** 5 × 10^4^ R1 cells/well were seeded onto six-well plates. On the next day, cells were co-transfected with 1 μg of the PY4-luciferase (i) or RGC-luciferase (ii) and 0.1 μg of Renilla luciferase plasmids. The luciferase levels were measured using the luciferase assay system (Promega) and normalized to the Renilla luciferase activity. **D** 5 × 10^4^ cells/well R1 cells were seeded onto six-well plates and co-infected mazEF or ΔPY4-mazF-mazE control viruses at an MOI of 7.5. Expression levels of the toxin (represented by the mCherry protein) and the antitoxin (represented by the GFP protein) were validated by western blot analysis. **E** Light and fluorescence microscopic examination of the infected cells at an MOI of 7.5.

The potency and ability of mazF, carried by adenoviruses, to selectively kill R1 cells, was evaluated and compared to the control virus having mazF but lacking the PY4 enhancer. Massive, dose-dependent cell death (7–80%) was induced in R1 cells upon infection with the Ad-PY4-SV40-mCheryMazF vector compared to the control virus (Ad-ΔPY4-SV40-mChery-MazF) (Fig. 2A). Cell viability, measured by the MTT assay, was ~43% when infected with Ad-PY4-SV40-mChery-MazF with a multiplicity of infection (MOI) of 3.75, and 92 and 94% when infected with the control virus (Ad-ΔPY4-SV40-mChery-MazF) or mCherry (pAd-CMV-mCherry), resp. (*p* < 0.01) (Fig. 2A). The specificity of gene expression mediated by PY4 was further confirmed by the luciferase activity assay (Fig. 2Ci). Finally, the detection of the mCherry fluorescent protein by western blot analysis showed, again, the specific transcriptional activation of the viral DNA (Fig. 2D).

### MazE activity depends on the genetic status of *p53*

The regulation of the antitoxin expression by the tumor suppressor gene, *p53*, is what makes this an innovative approach. The human ribosomal gene cluster (RGC) sequence is a cognate WT p53-responsive element located upstream of its target genes [23]. A Luciferase activity assay was used to verify the ability of WT p53 to bind the RGC sequence and induce luciferase transcription in R1 cells harboring WT p53 (Fig. 2Cii). Normally, WT p53 is constitutively degraded through the proteasome pathway, and therefore the addition of a stimulator is required. For this purpose, 5-fluorouracil (5FU), a known p53 chemical inducer, was chosen. The addition of 5FU (50 µM) to R1 RGC-luciferase transfected cells significantly increased luciferase activity by a factor of 5 (Fig. 2Cii).

Then we tested whether the antitoxin can inhibit the toxicity induced by mazF. R1 cells were co-infected with mazF and mazE at a ratio of 2:1 (Fig. 2B). The survival rates of the co-infected R1 cells were similar to those of R1 cells infected with the toxin only (52 ± 2% compared to 43 ± 2%, respectively).

Observation under fluorescent microscopy of the mCherry and GFP reporter genes, representing mazF and mazE, resp., confirmed the transcriptional activation of the viral DNA and provided a qualitative evaluation of the cytopathic effect (CPE) (Fig. 2E).

### TA dual system induces selective cell death in CRC cells

**Fig. 3:**
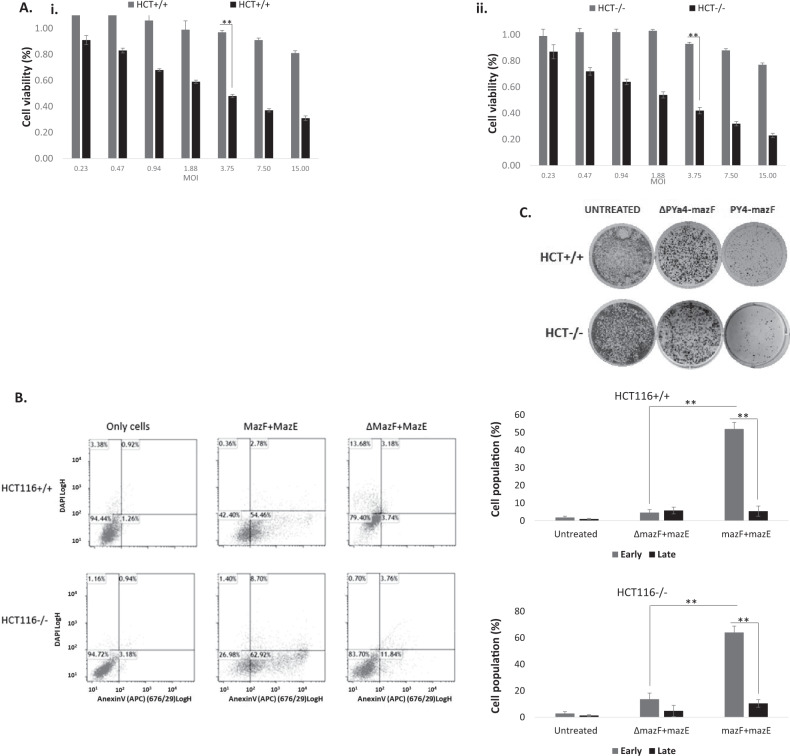
Eradication of CRC cells by mazEF-encoded adenoviruses. **A** 1 × 10^4^ cells/well HCT116^+/+^ (i) or HCT116^−/−^ (ii) cells were seeded onto 96-well plates and median dilutions of the mazEF or the ΔPY4-mazF-mazE control viruses were added to the cells, starting from an MOI of 15. Cell survival was measured by the enzymatic MTT assay 72 h after the infection and average values of triplicates, from representative experiment, were plotted. **B** 1 × 10^5^ cells/well were seeded onto 12-well plates. After 24 h, the cells were co-infected with mazEF or the ΔPY4-mazF-mazE control viruses at a 1:0.1 ratio and at an MOI of 7.5 for 72 h. Cell death was measured by flow cytometry after staining with Annexin V and DAPI dyes. **C** 5 × 10^5^ cells/well HCT116^+/+^ (i) or HCT116^−/−^ (ii) cells were seeded onto six-well plates and subsequently were co-infected with mazEF or the ΔPY4-mazF-mazE control viruses at a 1:0.1 ratio and at an MOI of 7.5, or left uninfected. After 7 h, cells were trypsinized, seeded at threefold dilutions, and subsequently incubated for 7 days. Surviving colonies were stained with 0.02% (v/v) crystal violet.

Co-infection at an MOI of 3.75 of HCT116^+/+^ (*RAS*^*mut*^*/p53*^*wt*^) and HCT116^−/−^ (*RAS*^*mut*^*/p53*^*mut*^) cell lines induced massive, dose-dependent cell death in both cell lines at 42 ± 1.7% and 48 ± 1.3%, resp. HCT116^−/−^ is a cell line derived from colorectal carcinoma in which two promotorless targeting vectors were used to disrupt sequentially the two p53 alleles [24]. This variant, kindly provided by Prof. Moshe Oren, is considered as null (Fig. 3A). In contrast, co-infection with ΔPY4-SV40-mCherry-mazF and RGC-SV40-IRES-mazE viruses showed significantly greater cell survival of both HCT116^+/+^ and HCT116^−/−^ cells at 93 ± 1.3% and 97 ± 1.4%, resp.

A colony formation assay showed a major difference in the number of colonies following mazF-mazE co-infection in comparison to untreated or ΔPY4-mazF-mazE co-infection (Fig. 3C). Furthermore, a selective cytotoxic effect was confirmed by flow cytometry using APC Annexin V and DAPI staining (Fig. 3B). This analysis confirmed that the TA system induced high levels of apoptosis of HCT116^−/−^ and HCT116^+/+^ target cells compared to control (~72% compared to ~16 and 57% compared to ~7%, resp.).

### Optimizing the balance between the toxin and the antitoxin

**Fig. 4:**
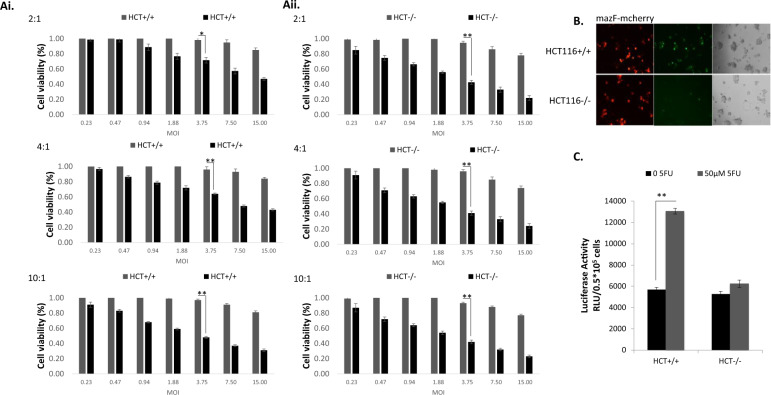
Finetuning of toxin–antitoxin dual system. **A** 1 × 10^4^ HCT116^+/+^ (i) or HCT116^−/−^ (ii) cells were seeded onto 96-well plates and median dilutions of the toxin–antitoxin or the control-antitoxin viruses, in three ratios (2:1, 4:1, and 10:1), were added to the cells, starting from 15 MOI on the next day. Cell survival was measured by the enzymatic MTT assay 72 h post infection and average values of three technical and two biological repeats are plotted. **B** Light and fluorescence microscopic examination of the co-infected HCT116^+/+^ and HCT116^−/−^ cells with toxin–antitoxin or control-antitoxin viruses at an MOI of 7.5. **C** 0.5 × 10^5^ cells/well HCT116^+/+^ (i) or HCT116^−/−^ (ii) cells were seeded onto six-well plates. On the next day, the cells were co-transfected with 1 μg of the RGC-luciferase and 0.1 μg of Renila luciferase plasmids. The luciferase levels were measured using the luciferase assay system and normalized to the Renila luciferase activity.

We then optimized the ratio between the constructs carrying the toxin and the antitoxin to enable the induction of a highly selective cytotoxic effect in malignant cells, while protecting normal cells. A series of MTT assays with different ratios of mazF:mazE (2:1, 4:1, and 10:1) were performed. Massive, dose-dependent cell death was observed in all cell lines (Fig. 4A).

We then focused on cells harboring both hyperactive KRAS and WT p53, since in those cells, both arms of the dual system can be activated (Fig. 4B, C). We hypothesized that reducing the antitoxin dose, while keeping the toxin constant, would lead to lower cell survival. Indeed, the survival assays showed higher survival rates of HCT116^+/+^ cells (48 ± 1.263% at a mazF:mazE co-infection ratio of 10:1, compared to 72 ± 3.7% at a rate of 2:1, at an MOI of 3.75). In contrast, in HCT116^−/−^ cells harboring truncated p53, no significant difference in cell survival was found even with increased doses (43 ± 2% at a mazF:mazE co-infection ratio of 10:1, compared to 43 ± 2.8% at a ratio of 1:0.5, at an MOI of 3.75). The selective antitoxin activation was confirmed by fluorescent microscopy, showing sufficient GFP expression in HCT116^+/+^ co-infected cells, compared to HCT116^−/−^ cells (Fig. 4B). Similarly, a luciferase activity assay showed selective enhanced activity due to the presence of induced WT p53 (Fig. 4C).

### The RRE enhances gene expression in cells that carry mutations of all Ras variants

**Fig. 5:**
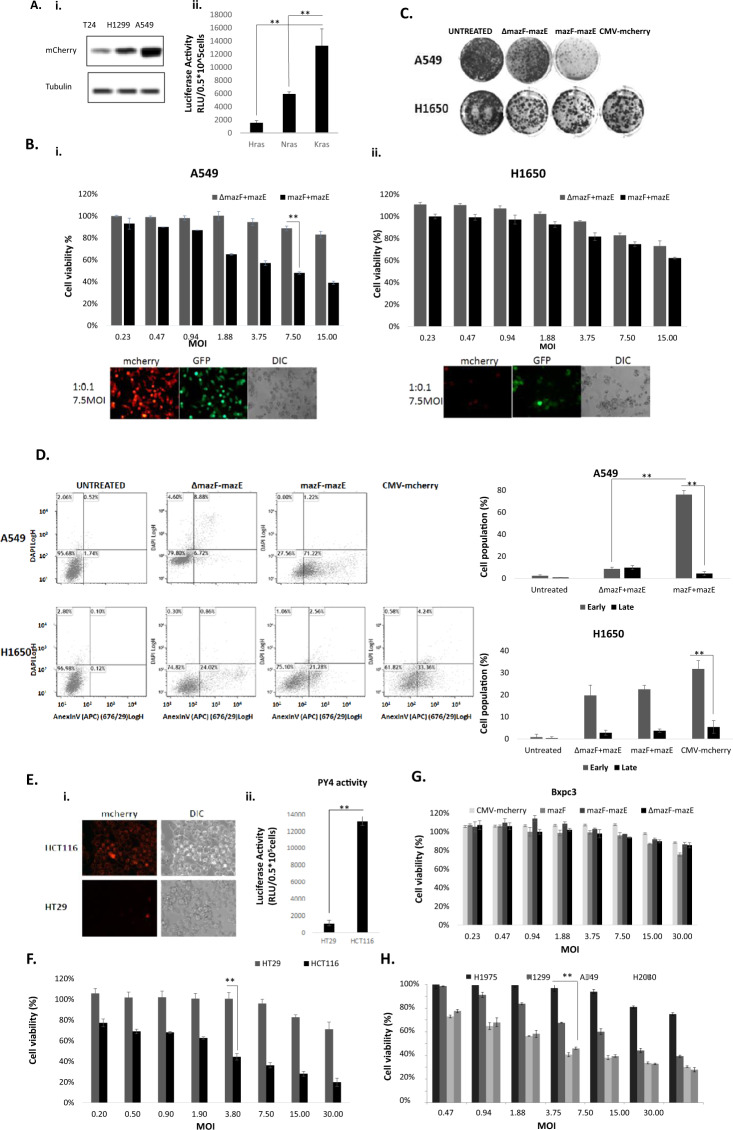
Eradication of lung cancer cells by recombinant toxin and antitoxin adenoviruses. **A** (i) 0.5 × 10^5^ cells/well H1299, A549, or T24 cells were seeded onto six-well plates. On the next day, the cells were co-infected with toxin–antitoxin or control-antitoxin constructs, at a 1:0.1 ratio and at an MOI of 7.5. Expression levels of the toxin (represented by the mCherry protein) and the antitoxin (represented by the GFP protein) were validated by western blot analysis. (ii) 0.5 × 10^5^ cells/well H1299, A549, and T24 cells were seeded onto six-well plates. On the next day the cells were co-transfected with 1 μg of the PY4-luciferase and 0.1 μg of Renila luciferase plasmids. The luciferase levels were measured and normalized to the Renila luciferase activity. **B** 1 × 10^4^ cells/well A549 (i) or H1650 (ii) cells were seeded onto 96-well plates, and median dilutions of the toxin–antitoxin or the control-antitoxin viruses, at a ratio of 1:0.1, were added to the cells, starting from an MOI of 15 on the next day. Cell survival was measured by the enzymatic MTT assay 72 h post infection. **C** 0.5 × 10^6^ cells/well A549 and H1650 cells were seeded onto six-well plates and subsequently co-infected with the toxin–antitoxin or the control-antitoxin viruses at a 1:0.1 ratio and at an MOI of 7.5, or left uninfected. After 7 h, the cells were trypsinized and seeded at threefold dilutions and incubated for 7 days. Surviving colonies were stained with 0.02% (v/v) crystal violet. **D** 1 × 10^5^ cells/well A549 or H1650 cells were seeded onto 12-well plates. On the next day the cells were co-infected with the toxin–antitoxin or the control-antitoxin viruses in 1:0.1 ratio at an MOI of 7.5 for 72 h. Cell death was measured by FACS analysis after staining with Annexin V and DAPI dyes. **E** Microscopic assesments of mazF and mazE expression which are represented by the mCherry and GFP, respectively. **F** Luciferase levels were measured using the luciferase assay system and normalized to the Renila luciferase activity in HT29 and HCT116 CRC cell lines (as described above). **G**, **H** Cells were seeded in 96-well plates and median dilutions of the mazF, CMV-mCherry, mazF-mazE or ΔmazF-mazE viruses, in 10:1 ratio, were added to the cells, starting from an MOI of 30 on the next day. Cell viability was measured by the enzymatic MTT assay 72 hours post infection and 50 μg 5FU exposure. An average value of three technical and two biological repeats (mean ± SD) are plotted. Statistical significance (**p < 0.01) was calculated by two-tailed Student’s t-test.

Three cell lines, including H1299 (lung cancer), A549 (lung cancer), and T24 (bladder cancer), harboring the NRAS, KRAS, and HRAS oncogene, resp., were transfected with the PY4- -Luciferase and Ranila plasmids. In a luciferase activity assay, all three hyperactive RAS variants were able to induce luciferase activity (Fig. 5Aii), as compared to control vectors. The most significant elevation of ~9-fold was detected in KRAS (A549)-transformed cells.

In addition, differential toxin expression was confirmed by western blot analysis of the conjugated reporter, mCherry and GFP (Fig. 5Ai).

### The TA dual system induces selective cell death of lung cancer cells

Selective, dose-dependent A549 (*KRAS*^*mut*^*/P53*^*wt*^) cell death was induced following mazF-mazE co-infection (7–60%, at an MOI of 0.23–15) (Fig. 5Bi). In comparison, co-infection of the same cells with the control vector (pAdEasy-ΔPy4-SV40mP-mCherry-MazF) and the RGC-SV40mP-MazE-IRES-GFP vector showed higher rates of survival (84–100% at the same MOIs). Moreover, H1650 (*KRAS*^*wt*^*/P53*^*wt*^) cells showed a mild reduction in cell viability with no differences between levels of TA and control-antitoxin co-infected H1650 cells (75 ± 2% and 73 ± 2%, resp.) at an MOI of 7.5 (Fig. 5Bii).

Death assays were conducted here as well. A549 and H1650 cells were co-infected with mazF-mazE vectors and control vectors at an MOI of 10. A colony formation assay showed a major difference between the number of A549 colonies upon mazF-mazE co-infection (367 ± 8 colonies) compared to untreated and control viruses (3759 ± 752 colonies and 2897 ± 289 colonies, resp. [Fig. 5C]). Moreover, following mazF-mazE co-infection, a reduction in the number of H1650 colonies was demonstrated, compared to untreated cells. However, a similar number of colonies survived mazF-mazE co-infection or ΔPY4-mazE co-infection (*p* < 0.77). In addition, infection with the pAd-CMV-mCherry control vector showed similar results (1 649 ± 396 colonies, Fig. 5C).

A selective cytotoxic effect was confirmed by flow cytometry (Fig. 5D). This analysis revealed massive apoptotic death of A549 cells upon treatment with the mazF-mazE TA system, compared to ΔPY4-mazF-mazE co-infected cells (~72% and ~16%, resp.). In contrast, no significant difference in cell death was observed between TA-treated H1650 cells and control co-infected cells (25 and 24%, resp.). Infection with pAdEasy-Py4-mCherry-MazF+pAdEasy-RGC-MazE-pIRES-GFP and with pAdEasy-CMV-mCherry led to similar results.

To confirm that the dual TA system is not active in non-transformed cells, it was evaluated and examined in several non-transformed cell types, including HCT116 and HT29 (Fig. 5E, F), as well as in Bxpc3 cells (Fig. 5G) and cell lines H1975, H1299, A549, and H2030 (Fig. 5H).

### The mazF-mazE TA system inhibits tumor growth in vivo

**Fig. 6:**
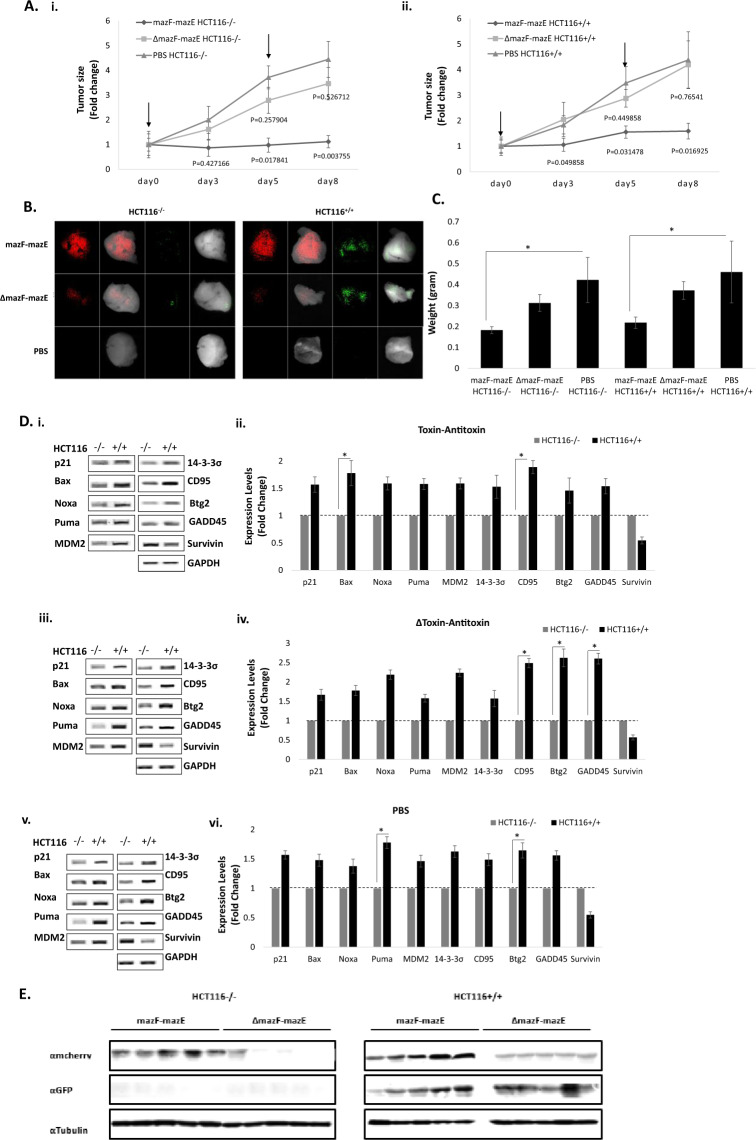
Inhibition of tumor growth in vivo. Tumors were formed in male nude mice by subcutaneous injection of 5 × 10^6^ cells/mouse HCT116^+/+^ or HCT116^−/−^ cells. Animals were treated with two toxin–antitoxin, control-antitoxin (2 × 10^9^–2 × 10^8^ PFU/mouse), or PBS, followed by intraperitoneal injection of 30 mg/kg 5FU. **A** Tumor size fold change normalized to initial tumor size at four time points in HCT116^−/−^ (i) and HCT116^+/+^ (ii) cells. The mean fold change values for each group are shown, and the standard deviation is represented by error bars for each measurement. The *p* values for the toxin–antitoxin group and control-antitoxin group compared to the PBS group are shown. Each bar represents the mean ± SD of a set of data determined from five mice. **B** Imaging was performed on fixed tumor sections with the Maestro imaging device. The red fluorescence dye represents the expression of the toxin and the green fluorescence dye represents the expression of the antitoxin. **C** Actual tumor weight was measured. **D** P53-dependent transcriptional activation following 5FU induction was validated by RT-PCR analysis of canonical target genes. Up- or down-regulation of canonical target genes was compared between HCT116^+/+^ and HCT116^−/−^ derived tumors in all treatment groups. **E** Expression levels of the toxin (represented by the mCherry protein) and the antitoxin (represented by the GFP protein) in the tumors were validated by western blot analysis.

Two intraperitoneal injections of the mazF-mazE vectors inhibited subcutaneous growth of both HC116^+/+^ and HCT116^−/−^ tumors compared to mice that were treated with ΔPY4-mazF-mazE control-antitoxin viruses or with phosphate-buffered saline (PBS) (Fig. 6A). By the end of the experiment, the overall volume of HCT116^−/−^ tumors was significantly smaller in animals that were treated with the mazF-mazE vectors (0.89 ± 0.03 cm^3^), compared to animals that were injected with the ΔPY4-mazF-mazE viruses (1.84 ± 0.05 cm^3^) or with PBS (2.3 ± 0.07 cm^3^), representing a 1.12-fold increase for the mazF-mazE group (*p* = 0.004), compared to a 3.46-fold increase for the ΔPY4-mazF-mazE group (*p* = 0.527) and 4.45-fold for the PBS group. HC116^+/+^-derived tumors that were treated with PBS showed similar results at a 1.59-fold change for the mazF-mazE group (*p* = 0.017) compared to 4.19-fold for the ΔPY4-mazF-mazE group (*p* = 0.765) and 4.38-fold for the PBS group (Fig. 6A).

During the above in vivo studies, mice were carefully monitored and no signs of toxicity were reported. At the end of the experiment, tumor weight was measured, resulting in a 2.5-fold difference between treated and untreated mice, supporting the above results (Fig. 6C). In the infected tumors, the expression of both the toxin and the antitoxin-conjugated fluorophores was monitored with the Maestro imaging CRi device (Fig. 6B). Moreover, western blot analysis confirmed the expression of the transgenes (Fig. 6E). In addition, reverse transcription PCR (RT-PCR) was performed on tumor cDNA and confirmed p53-dependent transcriptional activation due to up- or down-regulation of its canonical target genes, following 5FU induction (Fig. 6D).

## Discussion

RAS drugs target the disruption of regulator–effector interactions, the inhibition of membrane integration, the blocking of downstream effectors, the identification of synthetic lethal interactions, and RAS regulated metabolism, but have until now shown limited efficacy [25–27].

We are presenting a novel out-of-the-box generic platform for cancer therapy that is suitable to all tumor types. It is based on the profile of mutations and aberrant active pathways in tumors. Our suggested platform does not target specific single mutations, but rather the accumulation of complicated multi-factorial aberrant signaling pathways in cancer cells. At the same time, our developed MazEF protects normal cells from gene expression leakage that exists in biological systems, which is needed for routine activity of normal cells. The novelty of this platform lies in the selectivity, rapidity, and efficiency in eliminating tumor cells while sparing normal cells [16–19]. The regulation of the antitoxin expression by tumor suppressor gene *p53* makes this approach truly innovative.

We have so far developed four generations of this platform [16–18]. The first generation led to the selective elimination of CRC cells harboring hyperactive KRAS, using *PUMA* as the toxic agent [16–18]. In the second generation, the pro-apoptotic agent *PUMA* was replaced by a more potent moiety, an *E. coli* bacterial toxin (mazF) [19]. The third-generation construct (mazEF) addressed the low basal activity and the “leakiness” of all biological systems. In this system, a highly potent toxin, along with its natural antitoxin (mazE), has been introduced [19]. For the first time, it allowed active protection of normal cells, providing a significant advantage in the treatment of metastatic cancers.

One of the intriguing observations caused by the infection of cancer cells with mazEF was the increased cell death, as compared to mazF. It has been speculated that different levels of cell death could have been induced by the different stability of transcripts. Alternatively, the proximity of the MazE minimal promoter, and consequently, the transcription factors that are being recruited, could reinforce MazF’s minimal promoter activity [19]. This might cause higher toxin expression levels, and ultimately, increase cell death rate.

The fourth-generation construct became a dual system. Transcription regulation was tightened by separating the toxin and antitoxin into different cassettes, eliminating mutual transcription interference of MazF and MazE, and providing the ability to change their ratio according to the activation state of the RAS and p53 pathways.

The presented results describe a novel and generic platform that is suitable for any type of cancer depending on its tumor biology, driver mutations, and active pathways, while providing a substantial protective shield for normal cells.

This novel system exploits the expression of WT p53 to provide an additional layer of protection for normal cells, without harming the ability of the system to effectively kill cancer cells. In vitro, human CRC models have shown cell death of over 50% following mazEF co-infection (MOI of 3.75) in cells with both *RAS* and *P53* mutations (Fig. 3). A slight increase in cell survival was observed in cells that have mutated *RAS* but WT *p53*. This increase can be attributed to specific WT p53-dependent antitoxin activation. This increase was minor, confirming both the tight control of the therapeutic platform, and the correct balance between the two constructs, mazE and mazF. Even in cells with WT p53, this gene therapy approach is effective as the *RAS* construct dominates. Furthermore, the in vivo results in the CRC model supported our in vitro observations. Mice bearing either HCT116^+/+^ or HCT116^−/−^ derived tumors showed a significant inhibition of tumor growth following mazF-mazE treatment, compared to ΔPY4-mazF-mazE or PBS treated groups (Fig. 6).

The complexity and diversity of active pathways in cancer is enormous but it is minimized using our proposed treatment strategy. The inactivation of p53 is widespread in human malignancies and its loss-of-function can be a result of mutations, gene deletions, protein inhibition by specific viral proteins, increased expression of negative regulators, and alterations in upstream or downstream signaling pathways [28]. Mutant *p53* helps to potentiate the RAS pathway by interacting with specific transcriptional factors, one of which is Ets (Ets1 and Ets2), thereby facilitating the expression of mazF [29, 30].

The third-generation adenovirus platform-controlled mazE and mazF each with a different promoter. In the present study, the fourth generation of the platform is presented, in which identical promoters for both mazE and mazF were used. To control the balance between these two constructs different co-infection ratios were used. mazF favoring ratios were examined, including the natural 1:2 stoichiometry ratio that is observed in *E. coli*. Eventually, we chose a 10:1 mazF:mazE ratio, favoring the toxin. The use of this ratio supported our hypothesis that reducing the antitoxin dose, while keeping the toxin amount constant, increases efficacy.

The non-specific basal expression of the toxin as a result of the SV40 promoter’s leakiness has been balanced by regulated antitoxin expression. Our in vivo results showed that this leakage was almost completely blocked in WT p53 harboring HCT116^+/+^ tumors (4%, *p* < 0.3827). Therefore, the elimination of the toxic unregulated leakage by using the antitoxin might be interesting for clinical use.

Adenoviral vehicles have been introduced as an effective gene delivery tool in the past [31] due to their well-defined biology, genetic stability, effective gene transduction, and the ability to produce them at large scale [32]. Until now, no adenovirus-based cancer therapies have been approved by the American Food and Drug Administration (FDA) as they would not be effective treatment approaches. Most people have antibodies against adenoviruses and, therefore, developing a novel delivery tool that overcomes this limitation holds great promise.

In conclusion, the significant selective tumor regression and lack of toxicity hold promise for the development of a generic, novel, and effective therapy that is tailored to the tumor’s genetic profile and the downstream accumulation of all aberrant pathways. We are currently developing less immunogenic, potentially clinically applicable delivery systems based on small natural vesicles, such as exosomes, as they can directly target cancer cells through highly specific small antibody fragments that are over-expressed in most human cancer cells and rarely expressed on normal cells as, e.g., CD24 [22].

## Materials and methods

### Cell culture

Human lung cancer cell lines (H1299, H2030, A549, SHP77, H1650, and H1975) and a human pancreatic cancer cell line (BxPC3) were grown in Gibco™ RPMI 1640 medium (Thermo Fisher, USA, cat. no. 21875), supplemented with 5% (v/v) heat-inactivated fetal bovine serum (HI FBS) (Biological Industries, Israel), 1% (v/v) glutamine, 1% (v/v) penicillin/streptomycin. Human pancreatic cancer cell lines (Mia Paca2, Colo357, Panc1, and HEK-293), package cell lines (IEC18 and R1 KRAS), transformed rat enterocytes, and human CRC cell lines (HCT116, HCT116^+/+^, HCT116^−/−^, and HT29) were grown in Dulbecco’s modified Eagle’s medium (DMEM, Gibco™, supplied by Thermo Fisher, USA, cat. no. 11965118), supplemented with 5% (v/v) HI FBS, 1% glutamine, 1% penicillin/streptomycin. HCT116^+/+^ and HCT116^−/−^ cells were generated by Prof. Bert Vogelstein (The Howard Hughes Medical Institute, Johns Hopkins Oncology Center and Program in Human Genetics, Baltimore, MD, USA) [24] and kindly provided by Prof. Moshe Oren (Department of Molecular Cell Biology, Weizmann Institute of Science, Rehovot, Israel). Other cell lines were obtained from the ATCC. Human bladder cancer cell line T24 was grown in McCoy’s 5A Medium (Sigma, cat. no. M8403) and human bladder cancer cell line TCCSUP was grown in DMEM medium; both media were supplemented with 5% (v/v) HI FBS, 1% glutamine, 1% penicillin/streptomycin.

All cultures were grown at 37 °C under an atmosphere of 95% oxygen and 5% CO_2_.

### Bacteria strains

The following *E. coli* strains were used: DH5a (Stratagene, USA) for plasmid propagation and BJ5183 (Stratagene, USA) for the generation of recombinant adenovirus plasmid DNA.

### Construction of mazF and mazE encoding plasmids

We previously engineered the plasmids pAdEasy-Py4-mCherry-MazF, pAdEasy-CMV-mCherry [19], and pAdEasy-PY4-SV40-Luciferase [16]. Viral titer was evaluated by the CPE in an end-point dilution assay (data not shown).

### Western blotting

Co-infected cells were collected with a rubber policeman and washed twice in ice-cold PBS. Cell pellets were resuspended in lysis buffer (50 mM Tris-HCI at pH 7.5, 5 mM EDTA, 100 mM NaCl, 1% Triton, and 50 mM NaF) supplemented with a protease inhibitor cocktail (Sigma Aldrich, Israel, cat. no. 05892970001). An equal amount of protein (20 µg) from each lysate was analyzed by 10% SDS-polyacrylamide gels and subjected to electrophoresis. Proteins were transferred to Hybond-C membranes (Amersham, USA) in transfer buffer (25 mM Tris, 190 mM glycine, and 20% methanol), using a Trans-Blot® transfer system (BioRad, USA) at 300 mA for 1 h at room temperature (RT). Membranes were blocked with blocking buffer (5% skim milk in PBS containing 0.05% Tween-20 [PBS-T]) for 1 h. The membranes were incubated with 1:1000–1:5000 diluted primary antibodies (anti-GFP: Santa Cruz, USA, cat. no. sc-9996; anti-β-tubulin: Santa Cruz, USA, cat. no. sc-5274; anti-mCherry: Abcam, UK, cat. no. ab213511) for 1 h at RT. Membranes were then washed three times for 10 min in PBS-T, incubated with 1:2000–1:10000 diluted horseradish peroxidase-conjugated secondary antibodies (Jackson ImmunoResearch) for 1 h, and thoroughly washed again. Immune detection was performed using enhanced chemiluminescence.

### Colony formation assay

A549, H1675, HCT116^+/+^, and HCT116^−/−^ cells were seeded onto six-well plates (5 × 10^5^ cells/well). After 24 h, the cells were co-infected with either Ad-PY4-SV40-mCherry-MazF and Ad-PY4-SV40-MazE-IRES-GFP or with Ad-ΔPY4-SV40-mCherry-MazF and Ad-PY4-SV40-MazE-IRES-GFP, both at a MOI of 10, or they were left uninfected. After 7 h, the cells were trypsinized, seeded in threefold dilutions and incubated for 7 days at 37 °C, under an atmosphere of 95% oxygen and 5% CO_2_. Surviving colonies were fixed with 4% formaldehyde in PBS and stained with 0.02% (v/v) crystal violet.

### Luciferase activity assay

Transfections were performed by the calcium phosphate transfection method according to the standard protocols. Briefly, cells were seeded (3–5 × 10^5^) onto six-well plates. Twenty-four hours later, at 60–70% confluency, cells were co-transfected as follows: a mixture containing 1 μg DNA of pAdEasy-PY4-SV40-Luciferase or RGC-luciferase plasmid (the RGC sequence was amplified from RGC-mazE AdEasy system pShuttle vector with 5′-ATATATGCTAGCCCTGCCTGGACTT-3′ forward and 5′-ATATATAGATCTGATGGCCAGGCAAGTCC-3′ reverse primers. Then the RGC sequence was cloned into pGL3 promoter plasmid with *Nhe*I and *Bgl*II, upstream to SV40 promoter), 0.1 μg of Renilla luciferase plasmid (to evaluate transfection efficiency) (Promega, Madison, WI.cat. no. E223 Promega, Madison, WI, cat. no.E223), and 12.4 μl CaCl_2_ (2.5 M) in sterile DDW at a final volume of 100 μl was added (dropwise) to a solution containing 100 μl 2× HeBS (280 mM NaCl, 10 mM KCl, 1.5 mM Na_2_HPO_4_, 12 mM dextrose (glucose), 50 mM HEPES pH 7.05). After 15–30 min of incubation at room temperature, the mixture was sprinkled over the plate of cells.

Luciferase levels were measured after 72 h using the luciferase assay system (E1483, Promega, USA) and normalized to Renila luciferase activity according to the manufacturer’s instructions. Luminescence was measured by a LUMIstar® Galaxy luminometer (BMG Labtech, Germany).

### Detection of apoptosis

Cells were seeded onto 12-well plates (1 × 10^5^ cells/well) in complete medium and infected with the different adenoviruses (mazF-mCherry, ΔmazF-mCherry, RGC-mazE-pIRES-GFP, CMV-mCherry) at an MOI of 7.5 for 72 h. Annexin V (Annexin V, CF640R conjugate) was detected according to the manufacturer’s protocol (Biotium Inc., USA, cat. no. 4700). The cells were washed with PBS and then incubated in a solution of Annexin V-binding protein. The cells were subsequently analyzed by flow cytometry using the FACSCalibur™ platform (Becton Dickinson, USA), and the results were examined with CellQuest software (Becton Dickinson, USA) according to the manufacturer’s instructions.

### Cytotoxicity effect

Cells were seeded onto 12-well plates (1 × 10^5^ cells/well) in complete medium and infected with the different adenoviruses at an MOI of 10 for 72 h. Dead cells were detected by DAPI hydrochloride, a cell membrane impermeable nuclear dye, according to the manufacturer’s protocol (BioGems, USA, cat. no. 2879038). The cells were washed with PBS and then incubated in (5 mg/ml) DAPI 1:500 diluted solution. Ultraviolet nuclear staining was detected by flow cytometryCube6, Sysmex, Germany.

### Cell-viability assay

Exponentially growing cells were seeded onto 96-well plates (1 × 10^4^ cells/well). After 24 h, medial dilutions of recombinant adenoviruses were added. At 72 h post infection, the medium was replaced by fresh medium (100 μl/well) containing 1 mg/ml Thiazolyl Blue Tetrazoliam Bromide (MTT [Sigma, Israel, cat. no. M2128]) and the cells were incubated for 2–4 more hours. MTT-formazan crystals were dissolved by the addition of extraction solution (0.1 N HCl in absolute isopropanol). Absorbance at 570 nm and at a reference wavelength of 690 nm were recorded on an automated microplate reader (iMARK microplate absorbance reader, BioRadIsrael). The cell proliferation inhibition rate was expressed as the percentage absorbance relative to cells infected with pAdEasy-CMV-mCherry vector or to uninfected cells. The average of at least two independent experiments with triplicates was reported.

### Xenograft model in mice—in vivo tumor development evaluation

Male 6–8-week-old athymic nude mice (Hsd:Athymic Nude-Foxn1nu strain, purchased from Harlan Laboratories, USA) were housed in sterile cages and handled with aseptic precautions (*n* = 30). The mice were fed ad libitum. For testing the therapeutic potential of the TA dual system, exponentially growing HCT116^+/+^ and HCT116^−/−^ cells were harvested and resuspended at a final concentration of 5 × 10^6^ cells per 0.1 ml PBS per injection. The cells were injected subcutaneously at one site at the back of the mice. When tumors were palpable (~0.3–0.5 cm^3^), the mice were randomly divided into three groups (Table 1) and the treatment was initiated. Group allocation was done without investigator blinding. Each experimental group contained five mice. The viruses (1 × 10^9^ pfu) or PBS were administrated via two intraperitoneal injections with a 3-day interval between the injections. In all, 30 mg/kg fluorouracil (5FU, Israel, Sigma, cat. no. F6627) was injected intraperitoneally to all mice on the same day. The mice were weighed, tumor volume was measured with a caliper every 2 days starting from treatment onset, and the results were carefully plotted. Tumor volume was calculated as 4/3*π* × *a* × *b*^2^ with *a*, smaller length, and *b*, longer length of the tumor.

**Table 1:** List of primers.

Gene	Sequence 5′–3′	Gene	Sequence 5′–3′
Noxa F	AGAGCTGGAAGTCGAGTGT	hBax F	TGAGCAGATCATGAAGACAGGG
Noxa R	GCACCTTCACATTCCTCTC	hBax R	GCTCGATCCTGGATGAAACC
hPUMA F	TCAACGCACAGTACGAGCG	CD95 F	CCCTCCTACCTCTGGTTCTTACG
hPUMA R	GTAAGGGCAGGAGTCCCATG	CD95 R	TTGATGTCAGTCACTTGGCAT
GADD45A F	CTCAACGTCGACCCCGATAA	Btg2 F	CCAGGAGGCACTCACAGAGC
GADD45A R	ACATCTCTGTCGTCGTCCTCG	Btg2 R	GCCCTTGGACGGCTTTTC
P21 F	GGCAGACCAGCATGACAGATT	MDM2 F	CAGGCAAATGTGCAATACCAA
P21 R	GCGGATTAGGGCTTCCTCTT	MDM2 R	GGTTACAGCACCATCAGTAGGTACAG
14-3-3 sigma F	GCCTATAAGAACGTGGTGGGC	Survivin F	CCACCGCATCTCTACATTCA
14-3-3 sigma F	CCTCGTTGCTTTTCTGCTCAA	Survivin R	CAAGTCTGGCTCGTTCTCAGT
GAPDH F	AGCCTCAAGATCATCAGCAATG	GAPDH R	CACGATACCAAAGTTGTCATGGAT

At the end of the experiment, the animals were euthanized and their total tumor burden was excised and measured. MazF and MazE expression was monitored with a Maestro imaging device (Cambridge Research & Instrumentation, USA). The study was approved by the Institutional Review Board of Tel Aviv Sourasky Medical Center. Mice were purchased from Envigo, Israel.

### Total RNA extraction

Fifty to 100 mg tumor tissue was homogenized in 1 ml of TRIzol™ reagent (Thermo Fisher, cat. no. 15596026) using a homogenizer. In all, 0.5 mL of isopropanol (Bio-Lab, Israel, cat. no. I9516) was added to the aqueous phase and incubated for 10 min at RT. The samples were centrifuged for 10 min at 12,000 × *g* at 4 °C. The supernatant was discarded. The pellets were resuspended in 1 ml 75% (v/v) ethanol and centrifuged for 5 min at 7 500 × *g* at 4 °C. The supernatant was discarded and the RNA pellet was air dried for 5–10 min. The pellets were resuspended in 20–50 µl RNase-free water.

### Semi-quantitative RT-PCR

Reverse transcription was performed using 1 μg of total RNA with 400 ng/μl of random hexamer primer using the Verso cDNA Synthesis Kit and following the manufacturer’s instructions (Thermo Scientific, USA, cat. no. AB1453). Next, the cDNA was used in PCR amplification using the primers listed in Table 2. To compare the level of expression of different genes, the amount of cDNA in each sample was calibrated against the level of GAPDH mRNA.

**Table 2:** In vivo study design.

	Group 1	Group 2	Group 3
Ad-PY4-SV40-mCherry-MazF	+		
Ad-PY4-SV40-MazF-GFP	+	+	
ΔAd-PY4-SV40-mCherry-MazF		+	
PBS			+

### Statistical analysis

All experiments were performed at least in triplicates. Quantitative data were analyzed using the Student’s *t*-test. For all tests, *p* < 0.05 was considered significant. Data were expressed as the mean ± standard deviation (SD).
